# Testing Mediators of Youth Intervention Outcomes Using Single‐Case Experimental Designs

**DOI:** 10.1002/cad.20310

**Published:** 2019-09-11

**Authors:** Gemma G. M. Geuke, Marija Maric, Milica Miočević, Lidewij H. Wolters, Else de Haan

**Affiliations:** ^1^ Utrecht University; ^2^ University of Amsterdam; ^3^ McGill University; ^4^ University of Science and Technology/AMC

## Abstract

The major aim of this manuscript is to bring together two important topics that have recently received much attention in child and adolescent research, albeit separately from each other: single‐case experimental designs and statistical mediation analysis. Single‐case experimental designs (SCEDs) are increasingly recognized as a valuable alternative for Randomized Controlled Trials (RCTs) to test intervention effects in youth populations. Statistical mediation analysis helps provide understanding about the most potent mechanisms of change underlying youth intervention outcomes. In this manuscript we: (i) describe the conceptual framework and outline desiderata for methods for mediation analysis in SCEDs; (ii) describe the main aspects of several data‐analytic techniques potentially useful to test mediation in SCEDs; (iii) apply these methods to a single‐case treatment data set from one clinically anxious client; and (iv) discuss pros and cons of these methods for testing mediation in SCEDs, and provide future directions.

Single‐Case Experimental Designs (SCEDs) are within‐individual comparisons increasingly recognized as a valuable alternative for Randomized Controlled Trials (RCTs) (Kazdin, 2019) to test intervention effects in youth populations. In SCEDs, the symptoms of interest of one or several participants are tested regularly, for example, monthly, weekly, daily, and/or hourly, over a period of time depending on the design of a specific SCED study. Examples of SCED designs include (1) an AB design (baseline period A followed by an intervention period B), (2) A_1_B_1_A_2_B_2_ design (in which an intervention is withdrawn during A_2_ and again introduced during B_2_ period), and (3) the multiple baseline SCEDs in which clients are randomized to different lengths of a baseline period A before introducing an intervention phase B, making it possible to account for maturity effects in clients or passage of time. Please see Tate et al. (2016) and Barlow, Nock, and Hersen (2009) for a more complete overview of different SCEDs.

Given the heterogeneous nature of youth and family problems, in some cases SCEDs may be the only or the best possible way to investigate intervention outcomes, either because the psychological condition is rare (e.g., certain comorbidity) or because analyses on a group level would imply loss of information (i.e., finding no intervention effect while an effect is present in a certain subgroup) (Gaynor & Harris, 2008; Maric, Wiers, & Prins, 2012). Further, SCEDs can be used to test (novel) interventions prior to investigations in potentially demanding and costly RCTs (Jarrett & Ollendick, 2012; Norell‐Clarke, Nyander, & Jansson‐Fröjmark, 2011). SCEDs also offer a great opportunity to stimulate collaboration between research and practice, unifying research questions that emerge from youth clinical practice on the one hand, and, on the other hand, research methodology to test these questions on the level of a single client (Borckardt et al., 2008).

As mentioned earlier, these and other benefits of SCEDs are recognized nowadays. At the same time, several challenges remain, mainly related to design and quantitative analysis of SCED data. Presently, various data‐analytic techniques exist to test intervention outcomes (i.e., changes in one symptom over time) in SCEDs (for an overview, see, e.g., Manolov & Moeyaert, 2017): These techniques range from indices of improvement of the symptom scores between phases (e.g., the classic percentage of nonoverlapping data: Scruggs, Mastropieri & Casto, 1987), to regression‐based approaches that aim to model the time‐series data (e.g., Center, Skiba, & Casey, 1985). More recent developments in this field concern, for instance, integrating data of several subjects (e.g., hierarchical linear modeling (Heyvaert et al., 2017) and methods that allow for testing effects with fewer observation points per participant (Borckardt et al., 2008; Maric, de Haan, Hogendoorn, Wolters, & Huizenga, 2015). However, methods that are able to test simultaneous changes in more than one variable (symptom) over time have not received much attention in this field. Developing these methods could aid in discovering variables that are responsible for changes in core client outcomes, the so‐called “mediators”. Identification of mediators can improve youth interventions by identifying effective intervention components, and costs of interventions can be reduced by removing less potent intervention components (MacKinnon & Dwyer, 1993). In case of SCEDs, knowledge about individual clients’ mediators of treatment outcomes could inform treatment‐decision making and lead to a more evidence‐based youth practice (Maric, Prins, & Ollendick, 2015).

Therefore, the purpose of the current paper is to: (i) describe the conceptual framework and outline desiderata for methods for mediation analysis in SCEDs; (ii) describe the main aspects of several data‐analytic techniques potentially useful to test mediation in SCEDs; (iii) apply these methods to a single‐case treatment data set from one clinically anxious client; and (iv) discuss pros and cons of these methods for testing mediation in SCEDs, and provide future directions.

## Mediators of Intervention Outcomes: Definitions and Criteria

Intervention mediators are “mechanisms or processes through which an intervention might achieve its effects” (Kraemer, Wilson, Fairburn, & Agras, 2002, p. 878). There are many examples of studies that tested treatment mediators using large group designs (i.e., RCTs) in different youth populations (for an overview please see Maric, Prins, & Ollendick, 2015). Kendall and Treadwell (2007), for example, tested whether cognitive behavioral therapy influenced changes in negative cognitions, and whether these were, in turn, associated with changes in anxiety outcomes. In youth with ADHD, it was tested whether treatment acceptance and session attendance mediated family treatment outcomes (MTA Cooperative Group, 1999). Dekovic, Asscher, Manders, Prins, and van der Laan (2012) tested a sequence of two mediators: changes in parental competence were found to lead to changes in parenting behaviors which in turn led to changes in adolescent externalizing behavior following intervention. Thus, the main idea of including mediators in the study is that a certain intervention will produce changes in the mediator and that these changes will, in turn, affect intervention outcomes. This is a simplification of the goals of mediation analysis, and there are numerous considerations related to mediation analysis. For a more in‐depth treatment about why it is important to test for mediation, we refer the reader to the paper by O'Rourke and MacKinnon (2018).

**Figure 3.1:**
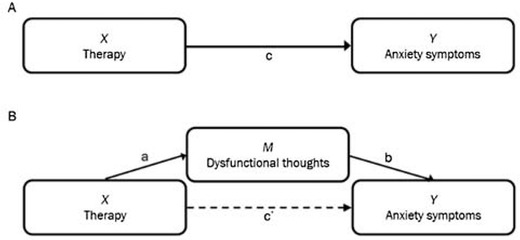
Hypothesized causal pathway for cognitive therapy, without and with mediator.

In more statistical terms, a mediating variable *M* is a variable that lies within the causal chain between an independent variable *X* and a dependent variable *Y* (MacKinnon, 2008) and represents the mechanism of change, as illustrated in Figure 3.1. Panel A indicates a hypothetical causal model in which therapy sessions (X) affect anxiety symptoms (Y).  In Panel B, this relationship is hypothesized to be mediated: the therapy sessions (X) are hypothesized to reduce dysfunctional thoughts (M), which in turn would relieve the young client of anxiety symptoms (Y). In this way, the effect of the therapy on anxiety symptoms should primarily take place through paths *a* and *b*, rather than through path c′. In the upcoming sections, we provide an overview of historical tests for mediation and the criteria that are necessary for understanding the framework in this paper.

## Testing for the Presence of Mediation

The model in Figure 3.1, panel A can be described and estimated using the first equation below. The model in Figure 3.1, panel B can be described and estimated using the second and third equation below.

(1) $$\hat{Y}=i_{1}+cX$$

(2) $$\hat{M}=i_{2}+aX$$

(3) $$\hat{Y}=i_{3}+c\prime X+bM$$

From these regression equations, several effects can be estimated using group‐level experimental data (Baron & Kenny, 1986; Judd & Kenny, 1981; MacKinnon, 2008). First, the so‐called total effect of the therapy on the symptom variable, noted by path *c* here, can be estimated. Second, the mediated or indirect effect of therapy on the symptom variable can be estimated. The indirect effect refers to the part of the effect that is transmitted through the mediating variable. The remaining effect of the therapy on the symptom variable that is not mediated by the mediator, is called the direct effect (path c′). The indirect effect can be computed by either taking the product of paths *a* and *b*, or by subtracting path c′ from path *c*; these two approaches lead to the same estimate of the indirect effect in linear models with no missing data (MacKinnon, Warsi, & Dwyer, 1995). Some of the first proposed approaches for testing for mediation consisted of the evaluation of whether the *c*, *a*, and *b* paths were significant and whether c′ was smaller than *c* (Baron & Kenny, 1986; Judd & Kenny, 1981). Note that these so‐called causal steps approaches do not provide an estimate of the numerical value of the indirect effect (MacKinnon, Lockwood, Hoffman, West, & Sheets, 2002). Furthermore, the requirement that the total effect be significant before testing for mediation is no longer used in mediation analysis because it is possible for an indirect effect to exist without a significant total effect, and approaches that focus only on the significance of the *a*‐ and *b*‐paths or their product have more power to detect mediated effects than methods that require a significant total effect and a direct effect smaller than the total effect (MacKinnon et al., 2002). Modern approaches for testing the significance of the mediated effect consist of constructing confidence intervals for the mediated effect and evaluating whether zero is in the interval. Methods for constructing intervals for the mediated effect that use critical values from the distribution of the product (MacKinnon et al., 2002) or that do not make any assumptions about the distribution of the mediated effect (e.g., the bootstrap; MacKinnon, Lockwood, & Williams, 2004) lead to the best statistical properties.

## Criteria for Establishing Mediation in Clinical Settings and Implications for SCEDs

Mediation models posit a causal hypothesis, that is, the hypothesis that a certain treatment causes changes in the mediator which in turn causes changes in treatment outcomes. To infer causal effects from data, certain criteria need to be established. Kazdin and Nock (2003) summarized criteria for causal inference specifically for mediation in youth clinical studies, of which we will focus on the following: (a) temporal precedence, (b) (strong) association, (c) specificity and (d) experiment. Note that these are in line with criteria for mediation of other authors in a more general intervention context, such as Kraemer et al. (2002), MacKinnon (2008), and Maric et al. (2012).

(a) The criterion of *temporal precedence* requires that it be possible to verify whether the change in the outcome variable indeed resulted from a preceding effect of the therapy on the mediator. Therefore, the data should demonstrate a change in the mediator between therapy phases, and this change should occur before the change in the outcome variable.
(b) The criterion of (*strong*) *association* refers to the strength of a relation between the variables. In group‐level mediation, the joint significance test (MacKinnon et al., 2002) can be used to establish whether the relation between the independent variable and mediator (*a* path) and the relation between the mediator and outcome (*b* path) are significant. However, in SCEDs, strong association cannot be measured using group‐level analysis methods or effect sizes, since these analysis methods require entirely different data structure and between‐subject comparisons. However, the relation between therapy on the one hand, and mediating variables on the other hand (i.e., path *a*) can be estimated using various statistical methods in SCEDs. For instance, data could demonstrate an association between therapy and the mediator if there is a significant difference in average level of scores of an individual on the mediator between phases, or if the trend of the scores on the mediator changes between phases. That is, if adolescents rate their coping skills higher on average (or increasingly higher) during the treatment compared to the baseline phase, this could be indicative of the treatment effect on coping skills. If this improvement in coping skills is then followed by an improvement in symptoms (by comparing scores of symptoms with coping skills at a previous time point), these findings are indicative of a nonzero *b* path, which together with a nonzero *a*‐path suggests mediation. Also, there is currently no clear way to compute the magnitude of the mediated effect using analysis methods for SCEDs which is why we will mainly refer to the joint significance of the SCED equivalents of the a‐ and *b*‐paths rather than the strength of the mediated effect.
(c) The *specificity* criterion requires that the change in the mediator is due to the treatment, and that the change in the outcome is due to the mediator. In group designs, this is established using a comparison of the treatment and control groups. In SCEDs, specificity can, for instance, be investigated by determining how and whether immediate change of the mediator appears after a change in phase, and whether changes in the mediator precede changes in the outcome. In multiple baseline designs, in which a small set of SCEDs are combined by alternating the length of the baseline phase, specificity can also be established when the timing of the effect does not depend on the length of the baseline phase. Establishing specificity in the relationship between the mediator and outcome is more challenging than establishing specificity in the relationship between the treatment and mediator because the values of the mediator are not manipulated in the experiment (Pirlott and MacKinnon, 2016) describe several methods for manipulating the mediator in order to make causal inferences about the relationship between the mediator and outcome; however, this is not always feasible nor ethical, and thus we will not entertain these approaches in the current paper).
(d) The *experiment* criterion requires all other causes to be ruled out, for instance, by testing the hypothesized causal path in an experimental design. Although a SCED is an experimental design, clinical‐psychological therapies can hardly be investigated in a true experimental setting, that is, a setting in which subjects are completely isolated from other influences, comparable to a laboratory setting. Clinical improvement could have other possible causes that cannot easily be ruled out, such as maturation and mood of the client. Maric et al. (2012) suggested as an alternative that, in therapy research, mediating variables can be compared with variables which are not intended to change during a specific treatment, that is, the so‐called nonmediators. If the paths that constitute the indirect effect (i.e., the equivalents of the *a*‐ and *b*‐paths in SCEDs) are stronger for the proposed mediators than for the proposed nonmediators, then this is additional evidence for mediation.

Note that we focused on the criteria Kazdin and Nock (2003) offered that can be assessed using a single dataset. In addition to these minimum criteria, elements such as *consistency* of evidence of mediation across studies and *plausibility* of the mechanisms of change according to theories are criteria for establishing mediation across studies and contribute to the credibility of the observed mediated effect (Kazdin & Nock, 2003). Finally, establishing a *gradient*, that is, that more of an independent variable or mediator results in more improvement on the dependent variable, is another criterion Kazdin and Nock offer (2003).

## Proposed Framework for Mediation in SCEDs

As mentioned earlier, most current data‐analytic techniques for SCEDs are concerned with tests of univariate outcomes (i.e., one variable at the time). However, these analyses cannot automatically be applied to investigate mediators of intervention outcomes in SCEDs. The framework for mediation analysis in SCEDs that we propose in this paper is a combination of the joint significance approach (MacKinnon et al., 2002) and the criteria described by Kazdin and Nock (2003). More specifically, our approach concerns the SCED equivalents of the *a* and *b* paths in group‐level mediation analysis (which is what makes it akin to the joint significance approach), and we use the Kazdin and Nock (2003) criteria that are possible to evaluate for a given path using existing SCEDs analysis methods. Note that we still include information about the significance of the SCED equivalent of the total effect (the so‐called *c* path) because this might be of interest for researchers, but a significant total effect is not a prerequisite for testing for mediation (Kenny, Kashy, & Bolger, 1998, p. 260; MacKinnon et al., 2002).

## Methods to Test Mediators of Interventions Effects in SCEDs

Testing potential mediators of therapy effects in SCEDs requires considering at least two variables measured repeatedly over the course of the study, that is, at least one potential mediator and one dependent variable, across multiple phases (phase represents the independent variable in this mediation model) in the same statistical analysis. Drawing from our discussion of criteria above, analysis methods should provide us with information on temporal precedence, specificity and associations between all variables in the single mediator model. To our knowledge, there is only one explicit approach to assess mediation in SCEDS: the method utilized in a study by Gaynor and Harris (2008). The existing method is qualitative in nature because it depends on visual analysis, and it does not conform to any formal framework for testing mediation. One of our goals in this paper is to adapt quantitative methods for SCEDs to an established set of criteria and procedure for testing mediation. The Gaynor and Harris (2008) method deviates from this goal; thus we apply this method to the example data set, but we report the procedure and findings in Appendix A in order to avoid digressing from the main message of the paper. In contrast, we would like to suggest a method that combines several existing analysis methods developed for SCED data, that is, combining the Tau‐U and piecewise regression analysis with cross‐lagged correlations. We briefly describe these three methods. Due to space constraints and the main focus of the paper being the theoretical framework for which the methods are repurposed, we will not explain each method in detail; instead, we provide references for the interested reader.

We identified two promising methods for testing the *a*‐path in mediation analysis in SCEDs: Tau‐U and piecewise regression analysis. Analysis methods for SCED in general can evaluate univariate time series with phases on multiple aspects, such as level, trend, and immediacy of effect (Kratochwill et al., 2013). *Level* can be conceptualized as the mean or median of the scores of a participant within a phase. *Trend* can be measured as the slope of a fitted regression line for the scores of a participant within a phase, indicating whether scores seem to be increasing or decreasing over time. A method can be used to evaluate *immediacy* of an effect if it demonstrates to what extent the effect of the phase variable on the score variable is immediate or gradual. For instance, for the first score of a new phase (e.g., B), piecewise regression estimates the difference between an estimate of the score based on the trend and level of the previous phase (e.g., A) and an estimate of that score based on trend and level of the new phase (e.g., B). A large difference could indicate an immediate effect of the introduction of therapy. The Tau‐U and the piecewise regression analysis are chosen because they offer information on level, trend and even immediacy of the effect simultaneously, along with a significance test for the change between phases.

In general, tests of between‐phase changes in level and between‐phase changes in trend can be used to establish the criterion of (strong) association. For instance, an improvement of the mean of a symptom variable indicates an association between the therapy (phase) and the symptom, that is, the total effect. Methods that provide information about the immediacy of an effect can be used to assess whether improvements were immediate after the introduction of a new phase (indicating specificity) and to assess whether most of the improvement in a symptom (outcome) variable took place after improvement in the mediating variable, thus indicating temporal precedence.

### Tau‐U

Tau‐U measures the between‐phase change in the level of the variable by quantifying nonoverlap (Parker, Vannest, Davis, & Sauber, 2011). Pairwise difference scores between all scores from one phase and all score from a previous phase are made. For instance, if we had 4 data points in one phase and 5 data points in another, we would have 4 × 5 pairwise difference scores. Using the sign of these difference scores, the number of difference scores that are positive, negative or tied can be computed. The percentage of nonoverlapping pairs is computed by subtracting the number of negative difference scores from the number of positive difference scores and dividing it by the total number of difference scores. Tau‐U can also be used to quantify the nonoverlap of scores *within* phases, which can be interpreted as the within‐phase trend. Data points are then compared to adjacent scores, such that in a phase with 4 data points, there would be 6 (i.e., 3 + 2 + 1) difference scores.

The criterion of strong association can be established using the estimates of changes in level. If there appears to be a trend in the baseline phase which already indicates clinical improvement of the client, the estimated between‐phase change in level can be corrected using the estimated baseline trend. For instance, there might be a significant increase in level between the two phases for a client while the trend of improvement is similar in the two phases, providing no indication that the improvement is related to the introduction of a therapy. Analysis employing Tau‐U in this paper can be replicated using the Tau‐U calculator at http://www.singlecaseresearch.org/calculators/tau-u (Vannest, Parker, Gonen, & Adiguzel, 2016).

### Piecewise Regression Analysis

Piecewise regression analysis, first proposed by Center et al. (1985), allows for estimation of the immediacy of change simultaneously with the level and trend (Manolov & Moeyaert, 2017). Ordinary Least Squares regression lines are fit to scores separately for each phase and the differences between regression lines per phase are quantified. For instance, in an AB‐design, the regression coefficients of the piecewise regression analysis provide estimates of the level of the first time point of phase A (i.e., the intercept), of the trend in phase A (the regression coefficient for a linear time variable), the change in level at the start of phase B (difference between the intercept of phase B and the predicted score if this would have been a score in phase A), and of the change in trend between the two phases (the difference in regression coefficients of the linear time variable between phases) (Manolov, Moeyaert, & Evans, 2016). Example code for piecewise regression analysis of an SCED data with an AB‐design is provided in a tutorial by Manolov et al. (2016).

### Cross‐Lagged Correlations

To our knowledge, this is the only suitable SCED analysis method for testing the *b* path in mediation analysis. With cross‐lagged correlation the following question could be tested in SCED: Are changes in negative cognitions associated with changes in anxiety symptoms or *vice versa*? The analysis allows for the tests of *temporal precedence* (e.g., Do negative cognitions change before anxiety symptoms change?) and (the direction of) the association between mediator and symptom variables (e.g., Are decreases in negative cognitions associated with decreases in anxiety symptoms?). Output of the analysis provides us with information about the *cross‐lagged correlations*—the measures of association between two time series—and the association between two variables is tested “forward” and “backward” over time (Borckardt et al., 2008). Note that cross‐lagged correlations have been historically considered a method for inferring “causal predominance,” that is, whether changes in variable 1 cause (precede) changes in variable 2 or vice‐versa, but that this method can yield spurious results about the causal predominance of two variables (Rogosa, 1980). In this application, we assume that changes in the mediator cause changes in the outcome, which subsumes assumptions that the causal order of variables in the single mediator model is correct and the assumption of no measured confounders of the relationship between the mediator and outcome. We use the cross‐lagged correlation between the mediator and the outcome as a proxy for the *b*‐path in the single mediator model. Cross‐lagged correlations can be computed using the Simulation Modeling Analysis software available at https://www.clinicalresearcher.org/software.htm

We describe a dataset used to illustrate the methods below.

## Case Example

The participant was a 9‐year‐old boy who met inclusion and exclusion criteria^1^ for a broader study investigating the additional value of cognitive therapy over and above exposure therapy for youth anxiety. The SCED included multiple assessments during the first treatment phase (B‐phase = exposure; EXP) followed by a C‐phase (exposure + cognitive therapy; EXP+CT) and a follow‐up phase, D. All phases had a duration time of 4 weeks. The study was conducted jointly by the University of Amsterdam, Developmental Psychology, and de Bascule in Amsterdam, Academic Centre for Child and Adolescent Psychiatry. Both youth and parents provided written informed consent allowing their data to be used for the purposes of scientific research. At pretreatment, the participant met criteria for separation anxiety disorder (Clinical Severity Rating [CSR]^2^ = 6), and generalized anxiety disorder (CSR = 6), assessed via administration of the Anxiety Disorders Interview Schedule for Children/Parents (ADIS‐C/P; Silverman & Albano, 1996).

For the purposes of the current study, daily assessments of client's anxiety symptoms and coping ability were obtained during all phases. The client rated two items daily (“How anxious did you feel today?” and “How well could you deal with your anxiety?”) on a 5‐point scale^3^ ranging from “not at all” to “very much.” Previous theory and research suggested that coping could be an important mediator of cognitive and exposure therapy outcomes (Hogendoorn et al., 2014; Prins & Ollendick, 2003), and the data was gathered to gain insight into one of the main goals of the broader study, namely: Whether changes in coping ability are associated with the presence of cognitive therapy (phase) and whether changes in anxiety symptoms (Anxiety; outcome variable) follow and are negatively associated with changes in coping ability (Coping; mediator). The total number of observations per phase was twenty‐six, thirty‐two, and fourteen for phases B, C, and D, respectively. For the purposes of this study, missing data points were deleted, thus resulting in a dataset useful only for illustrating the methods. The percentage of missing data was 14,5% and data were missing for two reasons: on some days the participants did not fill out the items, and on other days the participants reported no anxiety and; thus, there was no coping required. For the purposes of the current study this remains an interesting data set; however, the deletion of the missing values could result in inaccurate findings about the timing of therapy effects; thus one should not interpret the findings detailed here as the actual effect of this therapy.

## Results

Data were analyzed using methods as detailed in the methods section. First, we conducted a visual inspection of the data. Second, the Tau‐U, piecewise regression analysis, and cross‐lagged correlations were carried out.

**Figure 3.2:**
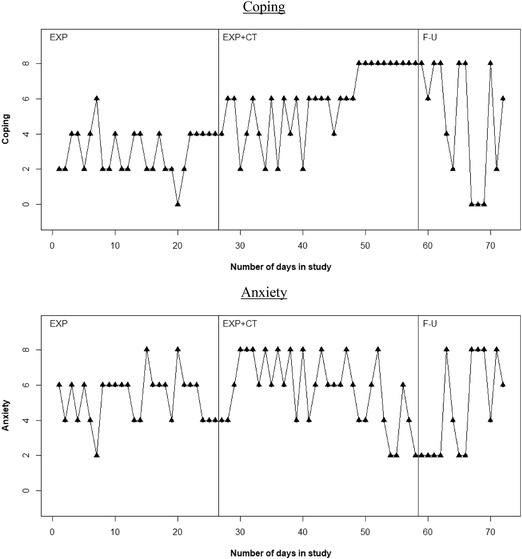
Graphical displays for the hypothesized mediator (Coping) and outcome (Anxiety) for the participant. No. of observations: phase B = 26, phase C = 32, phase D = 14.

For visual analysis, scores of Coping and Anxiety over time are plotted in Figure 3.2. These graphs can be used to inspect visually whether patterns of the scores (e.g., level or trend) have changed across phases, that is, whether the treatment in phase C and the absence of treatment in phase D could have caused a change in trend or level of Coping and Anxiety scores over time.

For both Anxiety and Coping, the scores mainly shift between two values throughout the EXP phase (respectively between 4 and 6 and between 2 and 4), with minimal shifts to values outside those two. Although scores of both variables somewhat stabilize on 4 at the end of this phase, these scores show no clear (improving) trend in the EXP phase. As the CT treatment is introduced at time point 27, Coping scores start to shift between 2 and 6 until time point 40, and after that show an increasing trend toward the highest score, 8. In that same time, Anxiety scores increase and remain high, between 6 and 8 until about time point 50, and show a decreasing trend after time point 50. Finally, as treatment is withheld in follow‐up phase, after 4 days of scores of 6 to 8, Coping scores start to vary between 2 and 8 and even 0 and 8, indicating a deterioration of Coping skills compared to the last part of the EXP+CT phase. A similar deterioration can be seen for Anxiety, as Anxiety scores vary between 2 and 8 again, also indicating an increase in Anxiety symptoms.

Overall, these observed changes in patterns of scores between phases could indicate that values of Anxiety and Coping are affected by the change in phases, which could therefore indicate an association between the variables and the phase, that is, the existence of the *a* and *c* paths in the mediation model. Regarding specificity, phase changes are visibly associated with changes in patterns of both Coping and Anxiety. For example, while Coping had stabilized for 5 days around the end of EXP, it started to shift between 2 and 6 from the start of the EXP+CT phase. The improvement of Coping and Anxiety seem to be related to the EXP+CT phase, as both the first phase and the follow‐up phase are much more variable. However, we cannot rule out other possible explanations of this change in variability in Coping and Anxiety between therapy phases, such as maturation and the mood of the client.

Regarding the association between mediator and outcome and temporal precedence, the scores of Coping of the participant seem to improve at the start of phase C (EXP+CT), while the improvement in Anxiety mainly takes place after the Coping has reached a stable high level (i.e., after time point 50). Also, as Coping is deteriorating in the follow‐up phase, so is Anxiety. To conclude, these initial findings of visual analysis suggest that the improvement of Anxiety level might be mediated by the improvement in Coping skills of the participant.

### Tau‐U Method Results

As noted in the Methods section, Tau‐U provides estimates of trend and between‐phase level change. Table 3.1 contains the results using Tau‐U.

**Table 3.1:** Results of the Analysis Using Tau‐U for Anxiety and Coping for the Participant

	Tau‐U	p‐Value	90% CI
*Trend Estimates*			
Coping (EXP)	0.065	0.64	[−0.165, 0.294]
Coping (EXP+CT)	0.534	<0.01*	[0.330, 0.739]
Coping (FU)	−0.275	0.17	[−0.605, 0.055]
Anxiety (EXP)	0.009	0.95	[−0.220, 0.239]
Anxiety (EXP+CT)	−0.280	0.02*	[−0.485, −0.076]
Anxiety (FU)	0.407	0.04*	[0.076, 0.737]
*Between‐Phase Level Difference*			
Coping (EXP vs. EXP+CT)	0.718	<0.01*	[0.465, 0.970]
Coping (EXP+CT vs. FU)	−0.092	0.62	[−0.399, 0.216]
Anxiety (EXP vs. EXP+CT)	0.160	0.30	[−0.093, 0.413]
Anxiety (EXP+CT vs. FU)	−0.221	0.24	[−0.529, 0.087]
*Corr. Between‐Phase Level Difference*			
Coping (EXP+CT vs. FU)	−0.683	<0.01*	[−0.991, −0.375]
Anxiety (EXP+CT vs. FU)	0.089	0.63	[−0.218, 0.397]

*Note*. Obtained using the Tau‐U web‐based calculator (Vannest et al., 2016) ^*^ *p* <.05, EXP = exposure phase (B), EXP+CT = exposure + cognitive therapy phase (C), FU = follow‐up phase (D)

Regarding trends, as was concluded in the visual inspection of the data, there is no clear trend in the EXP phase for both Anxiety (Tau‐U = .009, *p* = .95) and Coping (Tau‐U = 0.065, *p* = .64). In the EXP+CT phase, there is a significant trend for both variables, both in the expected direction, that is, a decreasing trend in Anxiety level and an increasing trend in Coping skills. In the follow‐up phase, Anxiety has a significant increasing trend and Coping has a nonsignificant decreasing trend.

For Coping, there is a significant increase in level between phase B and phase C (Tau‐U = 0.718, *p* < .01) and there is a nonsignificant decrease in level between phase C and phase D (Tau‐U = −0.092, *p* = .62), which turns into a significant decrease in level when corrected for the significant trend in phase C (Tau‐U = −0.683, *p* < .01). For Anxiety, there is a nonsignificant increase in level between phase B and phase C (Tau‐U = 0.160, *p* = .30) and there is a nonsignificant decrease in level between phase C and D (Tau‐U = −0.221, *p* = .24), which turns into a nonsignificant increase in level when corrected for the trend in phase C (Tau‐U = 0.089, *p* = .63), although it could be attributed to a floor effect.

Regarding strong association, the significant increase in level between phase C and B for Coping indicates that there is a strong association between Coping and EXP‐CT. Also, when corrected for the trend in the EXP‐CT phase, the change in level between phase D and C demonstrates a significant decrease of Coping skills after treatment is withheld. The results of the Tau‐U do not provide information about the immediacy of the change, which limits the information for the specificity and temporal precedence criteria. However, the trend estimates differ in direction between phases, which could suggest that the pattern of scores changes specifically due to the changes in phase. However, it would be necessary to test other plausible causes of change to establish specificity as defined by Kazdin and Nock (2003).

### Piecewise Regression Method Results

**Figure 3.3:**
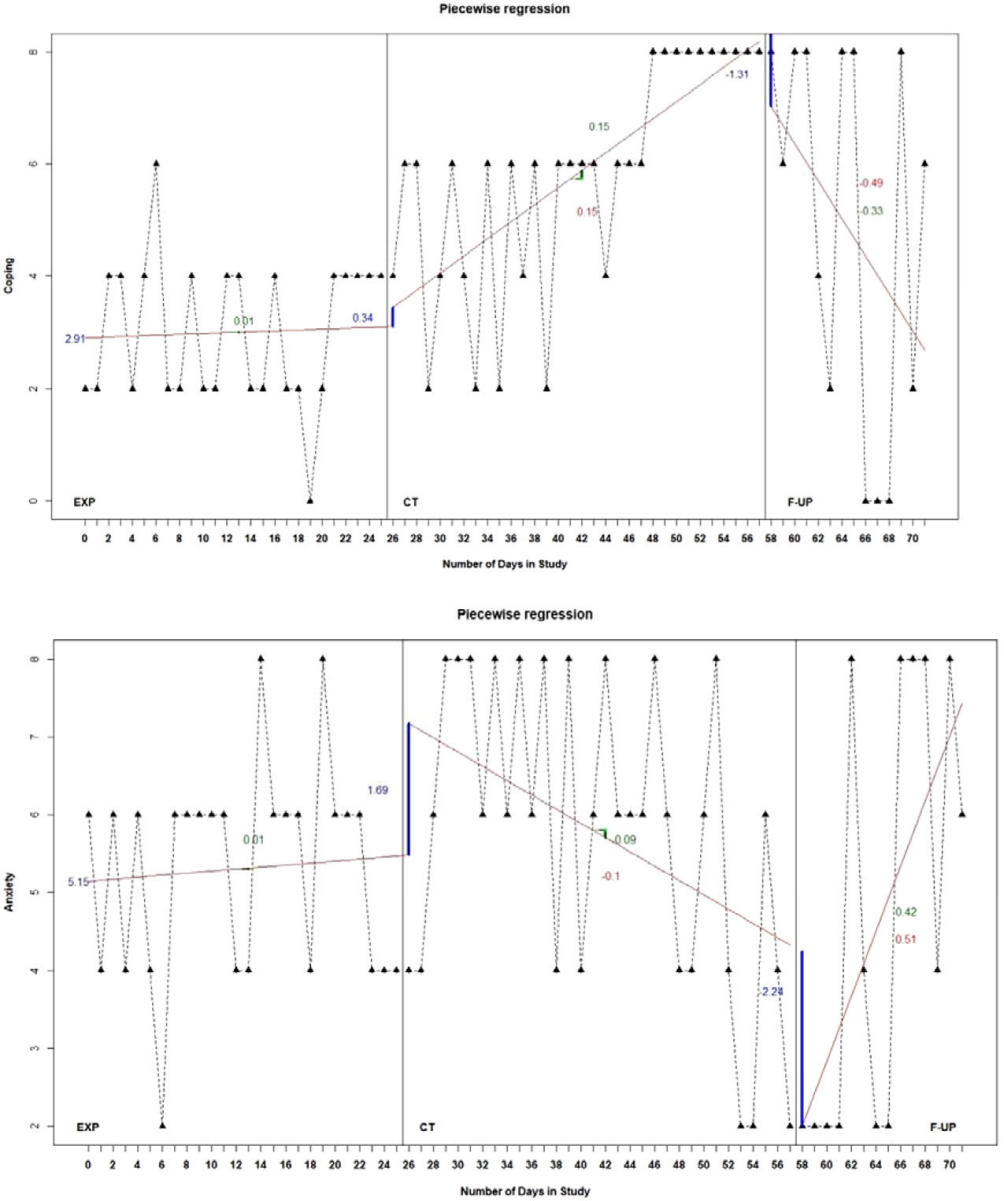
Graphical displays of the piecewise regression estimates for the hypothesized mediator (Coping) and outcome (Anxiety) of the participant. *Note*: The solid lines across phases indicate the estimated trend in the respective phase and the solid vertical lines at the start of each phase indicate the change in level between the respective phase and the previous phase. The blue numbers indicate the (change in) level, the green numbers indicate the within‐phase trend and the red numbers indicate the change in trend.

Results of the piecewise regression analysis can be seen in Figure 3.3 and Table 3.2. Due to the specific coding of the predictors, the regression coefficients of the piecewise regression analysis provide estimates of the level at the first time point in phase B (*intercept*), the trend in phase C (*time*1), the change in level at the start of phase D (*phase*), the change in trend between phases C and D (phase_time2), the change in level at the start of phase B (phase2), and the change in trend between phases D and B (phase2_time3) (Manolov et al., 2016).

**Table 3.2:** Results of Piecewise Regression Analysis for Anxiety and Coping of the Participant

	B	SE B	t‐Value	p‐Value
*Coping* [Link], [Link]				
Intercept	2.91	.52	5.57	<.001**
Time1	.01	.04	.21	.83
Phase	.34	.73	.47	.64
Phase_time2	.15	.04	3.28	.002**
Phase2	−1.31	1.30	−1.00	.32
Phase2_time3	−.49	.14	−3.38	.002**
*Anxiety* c,d				
Intercept	5.15	.62	8.31	<.001**
Time1	.01	.04	.31	.76
Phase	1.69	.86	1.95	.06
Phase_time2	−.10	.05	−1.99	.05
Phase2	−2.24	1.20	−1.86	.07
Phase2_time3	.51	.13	3.82	<.001**

*Note*. ^*^ *p* <.05; ^**^ *p* <.01.

^a^ *r^2^* = .154, *p* = .03.

^b^ *r^2^* = .319, *p* < .01.

^c^ *r^2^* = .637, *p* <.01.

^d^ *r^2^* = .348, *p* <.01.

Similar to the results of Tau‐U and the visual analysis, there is no clear trend in the EXP phase for both variables. For Coping, there is a slight increase in level at the start of the EXP+CT phase (B = 0.34, *p* = .64), followed by a significant positive change in trend, indicating an increasing trend (B = 0.15, *p* = .002). At the start of the follow‐up phase, the Coping score drops (B = −1.31, *p* = .32), followed by a significant negative change in trend, indicating a decreasing trend (B = −0.49, *p* = .002). For Anxiety, there is a nonsignificant increase in level at the start of phase C (B = 1.69, p = .06), followed by a marginally significant negative change in trend (B = −0.10, *p* = .05). Although the level of the scores at the start of phase D is quite below what was expected from the level of phase C (B = −2.24, p = .07), it is followed by a significant positive change in trend (B = 0.51, p <.001). The differences between the results obtained using piecewise regression and Tau‐U can be explained by the fact that piecewise regression provides relative estimates of trend, that is, a change in trend relative to the previous phase, rather than absolute estimates of trend.

All in all, the results of piecewise regression analysis provide moderate support for strong association between the therapy variable and the mediator (Coping). Similar to the results of the visual analysis, the immediate change‐in‐level estimate demonstrates that the level of Anxiety first reaches a high and stable level, followed by a decreasing trend. The immediate change‐in‐level estimate for Coping demonstrates a slight increase, followed by an increasing trend, which suggest that Coping is improving specifically due to therapy. These findings suggest that the increase in Coping skills precedes the clinical improvement in Anxiety level, which provides information for the temporal precedence criterion. Although the immediate change‐in‐level estimates of Anxiety are not in the expected direction, the findings support the hypothesis that Anxiety improves gradually due to therapy.

### Cross‐Lagged Correlation Results

As described in the Methods section, a cross‐lagged correlation is a measure of association between both time series of the mediator and the outcome variable (i.e., Coping and Anxiety) and can be used as a proxy for the *b*‐path. Results of cross‐lagged correlation can be found in Table 3.3. The column Lag indicates the number of days between the scores, that is, a lag of −01 indicates that each Anxiety scores was compared to the Coping score of 1 day earlier. Therefore, a negative lag means that the change in Coping preceded change in Anxiety.

**Table 3.3:** Cross‐Lagged Correlations Between Coping and Anxiety Across B and C Phase

Lag^a^	r
−05	−.18
−04	−.38**
−03	−.23*
−02	−.19
−01	−.16
0	−.56**

*Note*. Obtained using the Simulation Modeling Analysis (SMA) program (Borckardt et al., 2008).

a The standard number of lags provided in SMA is five.

^*^ *p* < 0.05, ^**^ *p* < 0.01.

As indicated in Table 3.3, Coping and Anxiety change concurrently (lag 0 correlation) or changes in Coping precede changes in Anxiety symptoms (significant lags −04 and −03). Unexpectedly, Anxiety scores are not significantly associated with Coping scores of 1 or 2 days in study earlier, but only at the same moment, or 3 or 4 days in study earlier. Therefore, our results with regard to the association between the mediator and the outcome, and the temporal precedence remain inconclusive.

## Discussion and Conclusion

In the current study we aimed to: (i) describe the conceptual framework and outline desiderata for methods for mediation analysis in SCEDs; (ii) describe the main aspects of several data‐analytic techniques potentially useful to test mediation in SCEDs; (iii) apply these methods to a single‐case treatment data set from one clinically anxious client; and (iv) discuss pros and cons of these methods for testing mediation in SCEDs, and provide future directions. The results are discussed in the light of criteria set for statistical mediation analysis (Judd & Kenny, 1981; Kazdin & Nock, 2003; MacKinnon, 2008). Overall, by using this approach on hypothetical data, there was some evidence that Coping could potentially mediate the effect of CBT on Anxiety for this participant. The initial visual analysis was useful to observe the variability between scores (corresponding to the *a* path) and to evaluate whether there was temporal precedence for changes in Coping and Anxiety (corresponding to the *b* path in the mediation model), but it had to be followed up with statistical analyses to determine whether the trends within phases and changes in level and trend between phases (corresponding to the *a* path in the mediation model) were in the hypothesized direction and significant. Crossed‐lagged analysis was helpful in determining the *b* path of mediation model.

Tau‐U provided significance tests to compare scores between phases, and more information about the strong association criterion. Tau‐U indicated that CT led to significant changes in Coping levels but not in Anxiety score levels, which provided additional support for the existence of the *a* path and no support for the existence of the *c* path.

Piecewise regression results indicated that there were significant changes in trend of Anxiety between the EXP+CT and follow‐up phases, thus providing moderate support for the existence of the *c* path. Results of piecewise regression also showed that there was a significant change in trend in Coping between the EXP and EXP+CT phases, thus providing evidence for the existence of the *a* path. Furthermore, the Coping scores improved before the Anxiety scores, thus providing some evidence for the criterion of temporal precedence.

Following these comparisons between the methods, evidence for mediation could be found in a conjunction of a significant between‐level differences on the mediator between the baseline and therapy phases (using Tau‐U), a significant changes in both level and trend in the hypothesized directions between baseline and therapy phases for the mediator (using piecewise regression), and temporal precedence in changes between mediator and outcomes (using cross‐lagged correlation).

One notable shortcoming of existing methods for SCEDs is that they could not be easily adapted to measure the strength of association between the mediator and outcome controlling for the independent variable (*b* path) and between the independent variable and outcome controlling for the mediator (c′ path). Thus, our inferences about the *b* path could only be based on whether the temporal precedence criterion was satisfied, and for this evidence we had to rely on visual analysis and cross lagged correlations, which then led to the question of expected *timing* of the changes in the mediator and outcome.

When using cross‐lagged correlations, it is unclear how many days should elapse after the introduction of EXP+CT before a change in the mediator, and how many days after that should there be a change in the outcome for the pattern to suggest an indirect effect of EXP+CT on Anxiety through changes in Coping. At the same time, this could be an issue related to the data sets in general in this type of research that is hard for analysis methods to accommodate to that. In this data set, Coping scores stabilize at a value of 8 near the end of EXP+CT; however, the scores in Anxiety are still oscillating and not showing a clear improvement in this time period. To establish temporal precedence of the changes in the mediator and outcome, we would need to find evidence that there was a change in both, and then observe whether this change follows the temporal sequence that matches our hypothesis and that occurs in a reasonable time frame. What counts as “reasonable” will vary between studies, populations, and therapies being tested.

The potential proxy for the *b* path described in this paper is the (lagged) correlation of Coping and Anxiety. However, this proxy is not equivalent to a partial regression coefficient that controls for the effect of the independent variable as in group‐level analyses. The analysis in this paper highlights the need for new methods for mediation analysis in SCEDs that can compute the *b* path and test whether it is statistically different from zero. One other issue that was not illustrated in our study per se, but is a common challenge for examining mediators (i.e., mechanisms of change during an intervention) in SCEDs is missing data. Even if one were to attempt multiple imputation of missing data (Peng & Chen, 2018), there are no studies that evaluated how much bias could be introduced in the results. Furthermore, even in group‐level analyses, the appropriateness of multiple imputation depends on the missingness mechanism (Rubin, 1976), and in clinical settings, it might be the case that data are missing not at random (MNAR; e.g., participants could fail to report their level of Anxiety on a given day because they were too anxious), which cannot be resolved by multiple imputation. In the example data set, there were two reasons for missing data: on some days the participants did not fill out the questionnaires, and on other days the participants reported no Anxiety and thus there was no Coping required. In the above analyses, we used listwise deletion because our goal was primarily to illustrate promising methods; however, it is not clear whether this practice is advisable when analyzing SCEDs.

In conclusion, the aims of this article were to illustrate the available methods for SCEDs that can be used for mediation analysis, and to highlight the developments that are still required for mediation analysis in SCEDs. While these methods still need to be tested in simulation studies, the application of these promising methods to the same real‐life data set illustrated some challenges that applied researcher encounter when testing mediated effects in SCEDs, and we discussed additional challenges that might arise with more missing data. Next steps for methodologists will be to develop methods that allow for the computation of the *b* path in the mediation model in SCEDs, so that all steps of the joint significance approach can be tested including the *b* path. In case of determining full or partial mediation on a single‐client level it would also be interesting to be able to calculate the c′ path, too. It further remains unclear whether the sum of the numerical estimates of indirect and direct effects would add up to equal the total effect (as in group‐level mediation analysis with continuous outcomes and no missing data), and whether we can also compute the indirect effect as c‐c′. Ideally, methods will be developed for SCEDs that perform better in group‐level mediation analyses, such as the distribution of the product and bootstrap confidence limits for the mediated effect (MacKinnon et al., 2002; MacKinnon et al., 2004). Mediation analysis in SCEDs is a very promising approach for evaluating how and why therapies work for individual clients, and the developments of methods for such analyses have only just begun.

## Notes

1 Inclusion criteria: ages between 8 and 18, primary anxiety disorder (except posttraumatic stress disorder or obsessive–compulsive disorder) according to DSM‐IV criteria, had not received protocolized evidence‐based CBT in the past half year, no use of selective serotonin reuptake inhibitors (SSRIs) at the moment of intake and treatment. Exclusion criteria: suicidal ideation, psychosis, selective mutism, IQ below 80, problems with drugs or alcohol.

2 The CSR scale consists of a 5‐point Likert scale (0–8 in steps of 2) and allows the clinician to evaluate the severity of each diagnosed condition. A score of 4 or above indicates the presence of a clinically significant disorder.

3 Same 5‐point scale was used here to avoid deviation from the scorings matrix was already used to during administration of ADIS‐C interview.

## Appendix A

### The Gaynor and Harris Approach

The Gaynor and Harris (2008) approach consists of decision criteria to establish mediation and both visual and statistical analyses to evaluate the change in the mediating variable and dependent variable. They proposed the following decision criteria:
Single‐participant assessment of treatment mediators requires documenting that the participant: (i) received treatment; (ii) improved during treatment; (iii) showed change on the proposed mechanism of action, which occurred at a reasonably expected time given the treatment protocol; and (iv) preceded a substantial portion of the clinical improvement. (2008, p. 375)

To illustrate similarities with the terminology used in this paper, step (ii) refers to an association between treatment and outcome (path c), step (iii) refers to an association between treatment and mediator (path a) and that this association is demonstrated by the data at the expected time (i.e., to estimate that is was specifically due to the treatment), and step (iv) refers to the criterion of temporal precedence. Step (i) does not receive explicit attention in our conceptual framework, and refers to the investigation of receipt of treatment by the participant.

#### Analysis Method

Regarding analysis, Gaynor and Harris did not provide an analysis protocol in their Methods section. The visual part of the analysis in the Results section consists of two elements. First, pretreatment and posttreatment levels of mediator and outcome variables are contrasted and compared to clinical cut‐off scores in order to establish associations. Second, the level and trends of several potential mediators and outcome variables are assessed visually in order to confirm these associations and establish specificity and temporal precedence. This visual analysis is similar to the approach used in this paper.

The visual analysis is supplemented with the ipsative *z*‐measure: The ipsative *z*‐scores is calculated using the following formula: ${z_{A}}_{j}=\frac{{X_{A}}_{j}-{\bar{X}}_{A}}{S_{A}}$ (Mueser, Yarnold, & Foy, 1991). This measure is used to compare each score (***X***) of a variable (***A***) at each time point (***j***) to the mean score (${\bar{X}}_{A}$) by dividing the difference by the standard deviation of the scores (***S***_***A***_). Subsequently, ipsative *z*‐scores are coded to reveal data patterns: a code of 1 is assigned if the symptom score was improving and a score of 0 if the symptom score was not improving. To establish the significance of the effect, critical difference values were used as proposed by Mueser et al. (1991). These values are calculated by computing 1,64***J***[1−***ACF***(**1**)]^(1/2), in which 1,64 is the one‐directional critical value for a *z*‐score, J is the amount of scores that are compared, and *ACF(1)* stands for the 1‐lag autocorrelation of the variable.

#### Demonstrating the Use of Ipsative *z*‐Scores Using an Example Dataset

The results for the ipsative *z*‐scores and the binary coding for Coping and Anxiety for case example can be found in Table A1. To reveal data patterns, the ipsative *z*‐scores were coded 1 when the score was equal to zero or higher or lower than the mean, depending on what was considered improvement for a given variable, and 0 in all other cases. That is, for Anxiety, zeros and negative scores were coded 1, and for Coping, zeros and positive scores were coded 1. Finally, the critical difference value of Anxiety is 1.64[2(1−.320)]^(1/2) = 1.91 and the value of Coping is 1.64[2(1−.468)]^(1/2) = 1.69. In case the absolute difference between two consecutive ipsative *z*‐scores equals or exceeds these critical difference values; this indicates a significant immediate change.

**Table A1:** Ipsative z‐Scores and Binary Coding for Each Score of the Participant

	Anxiety Level		Coping	
Time	Ipsative z	Coding	Ipsative z	Coding
1	0.31	0	−1.06	0
2	−0.70	1	−1.06	0
3	0.31	0	−0.25	0
4	−0.70	1	−0.25	0
5	0.31	0	−1.06	0
6	−0.70	1	−0.25	0
7	−1.72	1	0.56	1
8	0.31	0	−1.06	0
9	0.31	0	−1.06	0
10	0.31	0	−0.25	0
11	0.31	0	−1.06	0
12	0.31	0	−1.06	0
13	−0.70	1	−0.25	0
14	−0.70	1	−0.25	0
15	1.32	0	−1.06	0
16	0.31	0	−1.06	0
17	0.31	0	−0.25	0
18	0.31	0	−1.06	0
19	−0.70	1	−1.06	0
20	1.32	0	−1.88	0
21	0.31	0	−1.06	0
22	0.31	0	−0.25	0
23	0.31	0	−0.25	0
24	−0.70	1	−0.25	0
25	−0.70	1	−0.25	0
26	−0.70	1	−0.25	0
27	−0.70	1	−0.25	0
28	−0.70	1	0.56	1
29	0.31	0	0.56	1
30	1.32	0	−1.06	0
31	1.32	0	−0.25	0
32	1.32	0	0.56	1
33	0.31	0	−0.25	0
34	1.32	0	−1.06	0
35	0.31	0	0.56	1
36	1.32	0	−1.06	0
37	0.31	0	0.56	1
38	1.32	0	−0.25	0
39	−0.70	1	0.56	1
40	1.32	0	−1.06	0
41	−0.70	1	0.56	1
42	0.31	0	0.56	1
43	1.32	0	0.56	1
44	0.31	0	0.56	1
45	0.31	0	−0.25	0
46	0.31	0	0.56	1
47	1.32	0	0.56	1
48	0.31	0	0.56	1
49	−0.70	1	1.38	1
50	−0.70	1	1.38	1
51	0.31	0	1.38	1
52	1.32	0	1.38	1
53	−0.70	1	1.38	1
54	−1.72	1	1.38	1
55	−1.72	1	1.38	1
56	0.31	0	1.38	1
57	−0.70	1	1.38	1
58	−1.72	1	1.38	1
59	−1.72	1	1.38	1
60	−1.72	1	0.56	1
61	−1.72	1	1.38	1
62	−1.72	1	1.38	1
63	1.32	0	−0.25	0
64	−0.70	1	−1.06	0
65	−1.72	1	1.38	1
66	−1.72	1	1.38	1
67	0.48	0	−1.00	0
68	1.32	0	−1.88	0
69	1.32	0	−1.88	0
70	−0.70	1	1.38	1
71	0.48	0	−0.57	0
72	0.11	0	0.30	1

*Note*. The EXP phase ranges from time 1 to 26; the EXP+CT phase ranges from 27 to 58, and the FU phase ranges from 59 to 72.

The binary coding of Anxiety does not show a clear pattern of improvement: across all three phases, scores coded as clinical improvement remain shifting between 0 and 1 and, therefore, do not follow any clear pattern. The binary coding of Coping, however, does indicate an improving pattern: in the EXP phase, almost all of the scores are coded 0. In the EXP+CT phase, most scores (74%) are coded 1, especially in the second half of the phase. In the follow‐up phase, only a small majority (57%) of the scores are still coded 1. Note that the improving pattern of Coping merely indicates that the change in phase is associated with an increase in coping skills and does not provide enough evidence to indicate mediation. Regarding the critical difference values, none of the one‐time sequential differences equal or exceed the respective critical difference value, which indicates that there is no statistically significant immediate change (i.e., drop or increase) for Anxiety or Coping.

All in all, the coding provides some support for specificity for Coping, as most improved coded scores are in the EXP+CT phase. This also indicates that there is some support for an association between EXP+CT and Coping, but there is no indication of the size of that effect. However, as this measure demonstrates no clear pattern for Anxiety, it is not useful for comparing the timing of improvement between Anxiety and Coping and it provides no support for temporal precedence.

### Conclusion

The coding scheme used for ipsative *z*‐scores in the Gaynor and Harris (2008) method was useful for finding support for the existence of the *a* path, but not useful for the existence of the *b* path as the ipsative z‐scores method showed changes in one variable (Coping) when introducing a certain phase (EXP+CT). It was not possible to illustrate changes in coping preceding changes in anxiety. Further, none of the changes between consecutive time‐points were significant; however, this is not an issue unless it is expected that significant changes due to therapy occur immediately versus gradually. That expectation was not made in this study.
